# *Yersinia pseudotuberculosis* supports Th17 differentiation and limits de novo regulatory T cell induction by directly interfering with T cell receptor signaling

**DOI:** 10.1007/s00018-017-2516-y

**Published:** 2017-04-04

**Authors:** Maria Pasztoi, Agnes Bonifacius, Joern Pezoldt, Devesha Kulkarni, Jana Niemz, Juhao Yang, René Teich, Janina Hajek, Fabio Pisano, Manfred Rohde, Petra Dersch, Jochen Huehn

**Affiliations:** 1 Department Experimental Immunology, Helmholtz Centre for Infection Research, Inhoffenstrasse 7, 38124 Brunswick, Germany; 2 Department Molecular Infection Biology, Helmholtz Centre for Infection Research, 38124 Brunswick, Germany; 3 Central Facility for Microscopy, Helmholtz Centre for Infection Research, 38124 Brunswick, Germany

**Keywords:** Regulatory T cells, Th17 cells, *Yersinia pseudotuberculosis*, Intestinal infections, TCR signaling

## Abstract

Adaptive immunity critically contributes to control acute infection with enteropathogenic *Yersinia pseudotuberculosis*; however, the role of CD4^+^ T cell subsets in establishing infection and allowing pathogen persistence remains elusive. Here, we assessed the modulatory capacity of *Y. pseudotuberculosis* on CD4^+^ T cell differentiation. Using in vivo assays, we report that infection with *Y. pseudotuberculosis* resulted in enhanced priming of IL-17-producing T cells (Th17 cells), whereas induction of Foxp3^+^ regulatory T cells (Tregs) was severely disrupted in gut-draining mesenteric lymph nodes (mLNs), in line with altered frequencies of tolerogenic and proinflammatory dendritic cell (DC) subsets within mLNs. Additionally, by using a DC-free in vitro system, we could demonstrate that *Y. pseudotuberculosis* can directly modulate T cell receptor (TCR) downstream signaling within naïve CD4^+^ T cells and Tregs via injection of effector molecules through the type III secretion system, thereby affecting their functional properties. Importantly, modulation of naïve CD4^+^ T cells by *Y. pseudotuberculosis* resulted in an enhanced Th17 differentiation and decreased induction of Foxp3^+^ Tregs in vitro. These findings shed light to the adjustment of the Th17-Treg axis in response to acute *Y. pseudotuberculosis* infection and highlight the direct modulation of CD4^+^ T cell subsets by altering their TCR downstream signaling.

**Electronic supplementary material:**

The online version of this article (doi:10.1007/s00018-017-2516-y) contains supplementary material, which is available to authorized users.

## Introduction

The intestinal immune system requires an extremely tight control as it is constantly exposed to high loads of harmless foreign antigens such as microbiota and food, while at the same time it has to be ready to mount rapid and efficient immune responses against invading pathogens. Among these pathogens, enteropathogenic *Yersinia pseudotuberculosis* is known to initially infect the terminal ileum and Peyer’s patches, followed by an entering of mesenteric lymph nodes (mLNs). Infections with *Y. pseudotuberculosis* frequently result in the development of diarrhea, gastroenteritis, and mesenteric lymphadenitis [1, 2]. *Yersiniae* carry a broad range of virulence factors allowing interaction with immune cells and/or mediating immune evasion. Among others, they encode a type III secretion system (T3SS) on the pYV virulence plasmid, which enables translocation of effector proteins (Yops, Yersinia outer proteins) through a needle-like structure, referred to as injectisome [3]. Upon delivery into target cells, Yops (including YopE, H, J/P, K, M, O, and T) can interfere with intracellular signaling events, thereby manipulating key host cell functions such as cytokine secretion, actin cytoskeletal rearrangements, and phagocytosis [4, 5]. Recently, we could demonstrate that efficient Yop delivery into target cells is supported by the cytotoxic necrotizing factor CNFγ [6].

While innate immunity represents a well-characterized part of the immune response against *Y. pseudotuberculosis*, involving neutrophils, macrophages, dendritic cells (DCs), and natural killer cells [7–10], the role of the adaptive immune system in combatting invading *Yersiniae* is only incompletely understood. Besides studies underpinning the importance of CD8^+^ T cells in control of *Yersinia* infection [8, 11, 12], there are only few studies focusing on CD4^+^ T helper cell responses. These studies suggest the involvement of IFNγ-producing proinflammatory Th1 cells in protection against *Yersinia* [13], and report a capacity of CD4^+^ T cells in responding to *Y. pseudotuberculosis* superantigens in an MHCII-dependent manner [14]. A shift from immunoregulatory Foxp3^+^ regulatory T cells (Tregs) towards IL-17-producing proinflammatory Th17 cells has been reported for several enteropathogenic infections [15–18]. However, only little is known whether *Yersiniae* can directly modulate differentiation of CD4^+^ T cells, thereby favoring the establishment of infection [9, 19].

Here, we show that *Y. pseudotuberculosis* directly interacts with CD4^+^ T cells during the acute phase of infection and exemplify an involvement of Th17 cells and Tregs in the pathomechanism of disease. Using both de novo and in vitro T cell differentiation assays, we could demonstrate that T3SS-dependent modulation of T cells by *Y. pseudotuberculosis* results in a strongly impaired induction of Foxp3^+^ Tregs, while differentiation towards Th17 cells is highly supported. This immunological skewing of T cell differentiation is potentially mediated through the direct modulation of T cell receptor (TCR) downstream signaling pathways by the pathogen.

## Materials and methods

### Mouse strains

Foxp3^hCD2^ × Rag2^−/−^xDO11.10 (BALB/c), Foxp3^hCD2^ × CD90.1 (BALB/c), and Foxp3^hCD2^ (BALB/c) mice were bred and housed under specific pathogen-free conditions at the Helmholtz Centre for Infection Research (Braunschweig, Germany). BALB/c mice were purchased from Janvier. Gender- and age-matched mice were used in all experiments. Mice were housed and handled in accordance with recommendations of FELASA and the national animal welfare body GV-SOLAS guidelines. Experimental protocols were approved by the Lower Saxony Committee on the Ethics of Animal Experiments as well as the responsible state office (Lower Saxony State Office of Consumer Protection and Food Safety) under permit number 33.9-42502-04-13/1240.

### Antibodies and flow cytometry

Fluorochrome-conjugated anti-B220 (RA3-6B2), anti-hCD2 (RPA-2.10), anti-CD3 (17A2), anti-CD4 (RM4-5), anti-CD8 (53-7.3), anti-CD11b (M1/70), anti-CD11c (N418), anti-CD19 (6D5), anti-CD25 (PC61.5), anti-CD49b (DX5), anti-CD86 (GL1), anti-CD103 (2E7), anti-F4/80 (BM8), anti-Foxp3 (FJK-16S), anti-IFNγ (XMG1.2), anti-IL-10 (JES5-16E3), anti-IL-17 (TC11-18H10), anti-Ly6G (1A8), anti-MHCII (M5/114.15.2), anti-pERK1/2 (20 A), anti-RORγt (AFKJS-9), and anti-Ova-TCR (KJ1-26) antibodies were purchased from BioLegend, eBioscience, and BD. Intracellular Foxp3/RORγt and pERK1/2 stainings were performed according to the manufacturer’s instructions (Foxp3 Staining Buffer Set, eBioscience and Phosflow T Cell Activation Kit, BD, respectively). To determine the absolute number of living cells prior to flow cytometry analysis, propidium iodide (Sigma-Aldrich) was added, and cell number was determined using Accuri C6 Cytometer (BD). Dead cells were excluded based on the staining with the LIVE/DEAD Fixable Blue Dead Cell Stain (Thermo Fisher Scientific) and scatter properties. Cells were analyzed on LSRFortessa (BD) with Diva software v8.0.1 (BD), and data analysis was performed with FlowJo software v9.9.3 (TreeStar).

### Bacterial strains

The YPIII wild-type *Y. pseudotuberculosis* strain (Yptb-WT) [20] carrying the pIB1 plasmid was used throughout the study. The strains YP173 (Yptb-WT-Bla) and YP174 (ΔT3SS-Bla) were constructed by chromosomal integration of the YopE-β-lactamase (ETEM) fusion plasmid pSR47s-E-TEM1 with Yptb-WT and the T3SS-mutant ΔyscS strains [6]. Overnight cultures of *Yersiniae* strains were grown at 25 °C in Luria–Bertani broth medium (BD), washed, and diluted in PBS prior to infection. For in vitro co-culture experiments, bacteria were diluted 1:50 after overnight incubation, followed by incubation for 2 h at 25 °C and additional cultivation for 3 h at 37 °C. 50 μg/ml kanamycin (Sigma-Aldrich) was used for bacterial selection.

### Mouse infection

Female BALB/c mice (Janvier) aged between 6 and 7 weeks were subjected to fasting for 16 h prior to infection. Subsequently, mice were orally infected with 2 × 10^8^ Yptb-WT using a ball-tipped gavaging needle. 2 days p.i., the frequency of neutrophils in the peripheral blood of infected and non-infected mice was analyzed by flow cytometry, and significantly increased mobilization of neutrophils into peripheral blood of infected mice was taken as an indicator of a successful infection (data not shown). In general, body-weight loss and signs of severe illness of mice infected with 2 × 10^8^ Yptb-WT peaked at day 5–6. At indicated time points p.i., infected mice were analyzed or subjected to further experimental procedures.

### In vivo T cell differentiation assay

For adoptive transfer, single cell suspensions were generated from spleens and LNs of Foxp3^hCD2^xRag2^−/−^xDO11.10 mice. Before transfer, cells were labeled with the proliferation dye Cell Trace™ Violet (CTV, Thermo Fisher Scientific), and 4 × 10^6^ cells were injected in 100 µl PBS *i.v*. per recipient mouse. For induction of T cell differentiation, 20 µg Ova_323–339_ peptide was injected *i.v*. on two consecutive days, starting 1 day after adoptive T cell transfer. At day 3 after the first antigen application, cells were isolated from mLNs and stained for flow cytometric analysis. Intracellular cytokine staining was performed after restimulation with phorbol 12-myristate 13-acetate (PMA)/ionomycin for 2 h (10 and 500 ng/ml, respectively) and with 10 μg/ml Brefeldin A for additional 2 h at 37 °C (all from Sigma-Aldrich), followed by fixation and standard staining of surface markers.

### Field emission scanning electron microscopy (FESEM)

Total CD4^+^ T cells were enriched from spleens and LNs of BALB/c mice using CD4 (L3T4) MicroBeads and autoMACS separation (Miltenyi Biotec). CD4^+^ T cells were co-cultured with Yptb-WT for 1 h at MOI (multiplicity of infection) 100, followed by washing. Fixation was performed by 1 h incubation and washing with cacodylat buffer. Cells were placed on poly-l-lysine-coated cover slips, followed by fixation with 3% glutaraldehyde and washed with TE-buffer (0.02 M Tris, 0.001 M EDTA, pH 7.0), dehydrated with a graded series of acetone (10, 30, 50, 70, 90, 100%), and critical-point dried. After sputter coating with a gold film (appr. 10 nm), samples were analyzed using a Zeiss DSM 982 Gemini FESEM.

### β-Lactamase reporter assay

For in vivo analysis of Yop translocation, BALB/c mice were infected intragastrically with 2 × 10^9^ Yptb-WT-Bla. At day 3, single cell suspensions of mLNs were stained for cell surface markers and subsequently labeled with CCF4-AM, using the LiveBLAzer-FRET B/G Loading Kit (Thermo Fisher Scientific) for 1 h at room temperature in the presence of 1.5 mM probenecid (Sigma-Aldrich) and 50 μg/ml gentamicin (Sigma-Aldrich). To study Yop translocation in vitro, naïve CD4^+^ T cells were isolated from spleen and LNs of Foxp3^hCD2^ mice. Briefly, cells were stained with anti-CD25-APC and anti-hCD2-APC, followed by a depletion of APC^+^ cells using anti-APC MicroBeads (Miltenyi Biotec) and autoMACS separation system. Subsequently, CD4^+^ T cells were magnetically sorted as described before. The resulting naïve Foxp3^hCD2−^CD62L^hi^CD44^lo^CD25^−^ CD4^+^ T cells were co-cultured with Yptb-WT-Bla or ΔT3SS-Bla at an MOI of 10 for 1 h at 37 °C, washed twice with RPMI supplemented with 50 μg/ml gentamicin to eliminate bacteria. Subsequently, 2 × 10^6^ cells were labeled with CCF4-AM and analyzed by flow cytometry.

### Ca^2+^ signaling measurement

Total CD4^+^ T cells were enriched from spleens and LNs of Foxp3^hCD2^ mice via autoMACS separation. MACS-separated CD4^+^ T cells were co-cultured with Yptb-WT-Bla and ΔT3SS-Bla strains at MOI of 50 for 1 h at 37 °C and washed twice with gentamicin-containing RPMI. Subsequently, cells were stained for CD4 and Foxp3^hCD2^, loaded with 4 μg/ml Indo-1 AM cell permanent dye (Thermo Fisher Scientific) at 37 °C for 45 min in dark, followed by pre-decoration with 18 μg/ml anti-CD3-Biotin and 1 μg/ml anti-CD28-Biotin (both from BD). TCR crosslinking was induced by the addition of 40 μg/ml Streptavidin (Dianova) to the pre-warmed cell suspension. Ionomycin at 4 μg/ml concentration served as positive control providing maximum Ca^2+^ influx. The Ca^2+^ signal was measured in the respective gates of Tregs and naïve T cells by flow cytometry.

### pERK staining

Total CD4^+^ T cells were enriched from spleens and LNs of Foxp3^hCD2^ mice by autoMACS separation. MACS-separated CD4^+^ T cells were co-cultured with Yptb-WT-Bla and ΔT3SS-Bla strains at MOI of 50 for 1 h at 37 °C and washed twice with gentamicin-containing RPMI. Subsequently, cells were first stained with LIVE/DEAD Fixable Blue Dead Cell Stain and anti-CD4 antibody, followed by decoration with 10 μg/ml anti-CD3-Biotin and 5 μg/ml anti-CD28-Biotin antibodies for 15 min on ice. Crosslinking was induced by the addition of 10 μg/ml Streptavidin to the pre-warmed cell suspensions. At indicated time points, cells were fixed and permeabilized, followed by intracellular staining with anti-pERK1/2 and anti-Foxp3 antibodies overnight at 4 °C. Next, ERK phosphorylation was determined in the respective gates of live CD4^+^Foxp3 naïve T cells and CD4^+^Foxp3^+^ Tregs.

### In vitro T cell differentiation assay

Naïve CD4^+^ T cells were isolated from spleen and LNs of Foxp3^hCD2^ mice as described before. The resulting naïve Foxp3^hCD2−^CD62L^hi^CD44^lo^CD25^−^ CD4^+^ T cells were co-cultured with Yptb-WT-Bla and ΔT3SS-Bla at an MOI of 50 for 1 h at 37 °C. After the removal of bacteria by washing with gentamicin-containing RPMI, cells were cultured under Th0, Th1, Th17, or Treg-polarizing conditions. For Treg cultures, 5 × 10^5^ cells/well were cultured on 96-well round-bottom plates in RPMI supplemented with 10 ng/ml IL-2 (R&D), 5 ng/ml TGF-β1 (R&D), 50 μg/ml gentamicin, and anti-CD3/CD28 Dynabeads Mouse T Activator (Thermo Fisher Scientific) at 1:1 ratio. Frequency of Foxp3^hCD2+^ cells was determined 4 days later. For Th17 culture conditions, 2 × 10^6^ cells/well were cultured in 24-well plates coated with 3 μg/ml anti-CD3 (BioLegend) and 5 μg/ml anti-CD28 (eBioscience) in IMDM supplemented with 2 ng/ml TGF-β1, 30 ng/ml IL-6, 10 ng/ml IL-1β, 5 μg/ml anti-IL-2, 20 ng/ml TNF-α (all from BioLegend), 10 μg/ml anti-IFN-γ (BioXCell), and 50 μg/ml gentamicin for 4 days. Then, cells were replated in fresh medium and cultured without TCR stimulation for two additional days. On day 6, frequency of IL-17^+^ cells was determined after restimulation and fixation as described before. For Th0 and Th1 cultures conditions, 2 × 10^6^ cells/well were cultured in 24-well plates coated with 2 μg/ml anti-CD3 and anti-CD28 in IMDM containing 50 μg/ml gentamicin. The Th1 culture medium was supplemented with 20 ng/ml IL-12 (PeproTech) and 10 μg/ml anti-IL-4 (BioLegend), whereas cells cultured under Th0 conditions did not receive any cytokines or neutralizing antibodies. Cells were replated in fresh medium on day 2 and cultured without TCR stimulation for three additional days. On day 5, frequency of IFN-γ^+^ cells was determined after restimulation and fixation, as described before.

### Statistical analysis

Group sizes were estimated according to a presumed standard deviation (SD) and an expected type I error of <0.05. The sample size was adjusted, if required, based on initial results. For all figures, each data point represents a single mouse if not stated otherwise. For comparison of unmatched groups, two-tailed Mann–Whitney statistical test was applied. The comparison of more than two groups was performed by one-way ANOVA followed by Bonferroni’s post-test. All data are presented as mean or mean ± SD, and *p* values < 0.05 are considered as significant (*p < 0.05; **p < 0.01; ***p < 0.001; ****p < 0.0001). Prism software (GraphPad, La Jolla, CA, USA) was applied for all statistical analyses and graphs.

## Results

### Acute *Y. pseudotuberculosis* infection impairs de novo Treg induction and favors Th17 differentiation

**Fig. 1:**
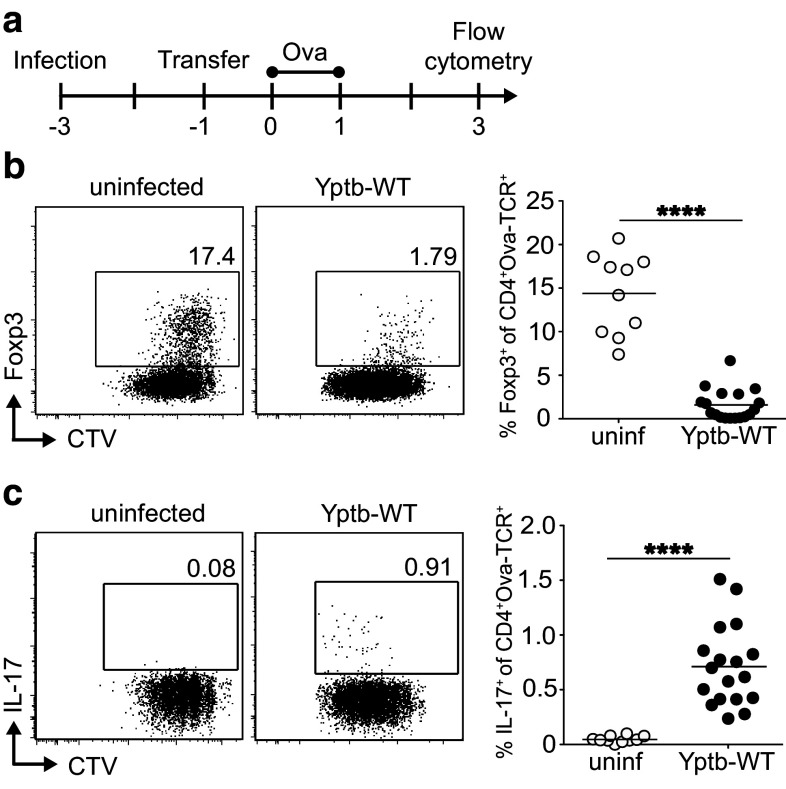
Abrogation of de novo Treg induction and increased Th17 differentiation during acute *Y. pseudotuberculosis* infection. **a** BALB/c mice were infected with 2 × 10^8^ Yptb-WT, and received 4 × 10^6^ CTV-labeled cells from Foxp3^hCD2^xRag2^−/−^xDO11.10 mice 2 days p.i. Uninfected mice were taken as controls. On the following 2 days, recipients were immunized via *i.v*. injection of Ova_323-339_ peptide and analyzed on day 3 after the first immunization by flow cytometry. **b, c** Representative *dot plots* show expression of Foxp3 **b** or IL-17 **c** over cell division (as indicated by loss of CTV) on adoptively transferred CD4^+^Ova-TCR^+^ T cells in mLN from Yptb-WT-infected mice or uninfected controls. *Numbers* indicate frequencies of cells in gates. *Scatter plots* summarize frequencies of Foxp3^+^ (**b**) or IL17^+^ (**c**) cells among adoptively transferred CD4^+^Ova-TCR^+^ T cells from indicated groups. Data are pooled from three independent experiments, each with 3–5 mice per group. ****p < 0.0001

The role of immunoregulatory Foxp3^+^ Tregs and proinflammatory Th17 cells in combatting acute *Yersiniae* infections is only incompletely understood [9, 19]. Thus, we first assessed whether acute infection with *Y. pseudotuberculosis* has an effect on the peripheral de novo generation of these two opposing T cell subsets. Since gut-draining mLNs are among the first target organs of *Y. pseudotuberculosis*, we decided to focus on T cell differentiation events taking place within mLNs. Thereto, BALB/c mice were infected sublethally with wild-type *Y. pseudotuberculosis* (Yptb-WT). At day 2 post infection (p.i.), shortly before the infection reaches its peak, TCR-transgenic, ovalbumin (Ova)-specific naïve Foxp3^−^CD4^+^ T cells were labeled with a cell proliferation dye and adoptively transferred into infected mice, while uninfected recipient mice served as controls (Fig. 1a). Subsequently, adoptively transferred naïve T cells were primed by systemic application of Ova peptide via the *i.v*. route on two consecutive days as reported previously [21]. At day 3 after the first antigen application, proliferating Ova-specific T cells within mLNs of uninfected and Yptb-WT-infected mice were analyzed by flow cytometry (Fig. 1b, c). As expected from previous observations [21], a high frequency of de novo-induced Foxp3^+^ Tregs among adoptively transferred T cells was observed in mLNs of uninfected mice. Importantly, de novo Treg induction was dramatically reduced in mLNs of mice infected with Yptb-WT (Fig. 1b). In parallel, the frequency of IL-17-producing cells among adoptively transferred Ova-TCR^+^CD4^+^ T cells, albeit being at a low level, was significantly higher upon infection with Yptb-WT when compared to uninfected controls (Fig. 1c), in line with a significant increase in IL-17-producing endogenous CD4^+^ T cells at day 8 p.i. (Supplementary Fig. S1). In contrast to this increase in IL-17-producing T cells, the frequency of IFN-γ- and IL-10-producing endogenous CD4^+^ T cells remained unchanged during the first 12 days p.i. (Supplementary Figure S1), further supporting the important contribution of Th17 cells to the control of *Y. pseudotuberculosis* infection [22]. Together, these results indicate that acute *Y. pseudotuberculosis* infection shifts the immunological balance from immunoregulatory towards proinflammatory T cells, thereby favoring the establishment of a protective, inflammatory environment in the intestine.

### *Yersinia pseudotuberculosis* infection alters composition of the DC compartment

**Fig. 2:**
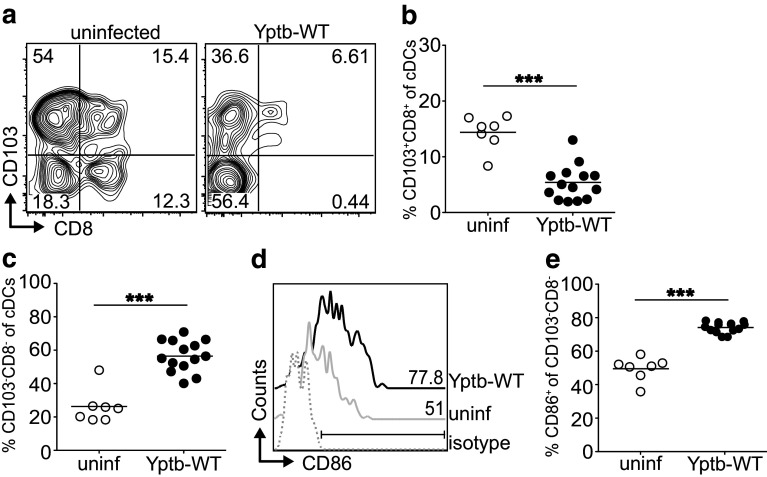
Acute *Y. pseudotuberculosis* infection results in decreased frequencies of tolerogenic CD103^+^CD8^+^ DCs, and expansion of proinflammatory CD103^−^ DCs. BALB/c mice were infected with 2 × 10^8^ Yptb-WT, and on day 5 p.i. cDC subsets within mLNs were analyzed by flow cytometry. Uninfected mice were taken as controls. **a** Representative *dot plots* depict CD8 and CD103 expression of Lin^−^(CD3^−^CD19^−^CD49b^−^B220^−^)CD11c^hi^MHCII^hi^ cDCs in mLN from Yptb-WT-infected mice or uninfected controls. *Numbers* indicate frequencies of cells in gates. **b, c** *Scatter plots* summarize the frequencies of CD103^+^CD8^+^ cells (**b**) and CD103^−^CD8^−^ cells (**c**) among cDCs from indicated groups. **d** Representative histograms depict CD86 expression of CD8^−^CD103^−^ cDC subset from indicated groups. The region was set according to isotype control staining, and *numbers* indicate frequencies of CD86^+^ cells. **e** *Scatter plot* summarizes frequencies of CD86^+^ cells among CD8^−^CD103^−^ cDC subset from indicated groups. Data are pooled from two independent experiments, each with 3–7 mice per group. ***p < 0.001

It is widely accepted that DCs play a key role for de novo induction of both Foxp3^+^ Tregs [21, 23] and Th17 cells [24]. Since gastrointestinal inflammation can strongly modify the DC compartment within mLNs, resulting in reduced tolerogenic properties of DCs [25], we next aimed to explore whether DC subsets were substantially altered within mLNs during acute infection with *Y. pseudotuberculosis*, and might contribute to the observed shift from Tregs to Th17 cells. At day 5 p.i., the overall frequency of MHCII^hi^CD11c^hi^ conventional DCs (cDCs) was strongly reduced in mLNs of mice infected with Yptb-WT (Supplementary Fig. S2a, b). However, absolute numbers of cDCs within mLNs did not change significantly when comparing infected mice to uninfected controls (Supplementary Fig. S2c). Nevertheless, we could observe a substantial alteration among specialized subsets within the cDC compartment. Tolerogenic CD103^+^CD8^+^ cDCs, known to be involved in de novo Treg induction [21, 23, 26, 27], were strongly reduced upon infection with Yptb-WT, whereas CD103^−^CD8^−^ cDCs, which have been described to be responsible for priming of Th1 and/or Th17 cells [24], were significantly expanded (Fig. 2a–c). Importantly, CD103^−^CD8^−^ cDCs displayed a strong increase in CD86 expression upon infection with Yptb-WT (Fig. 2d, e), reflecting their activated phenotype. Taken together, acute *Y. pseudotuberculosis* infection strongly affects the cDC compartment, and leads to a partial contraction of tolerogenic CD103^+^CD8^+^ cDCs, which might be responsible for the observed reduction of de novo Treg induction, while the expansion of CD103^−^CD8^−^ cDCs supports our initial observation of a shift towards Th17 differentiation.

### *Yersinia pseudotuberculosis* directly targets CD4^+^ T cell subsets

**Fig. 3:**
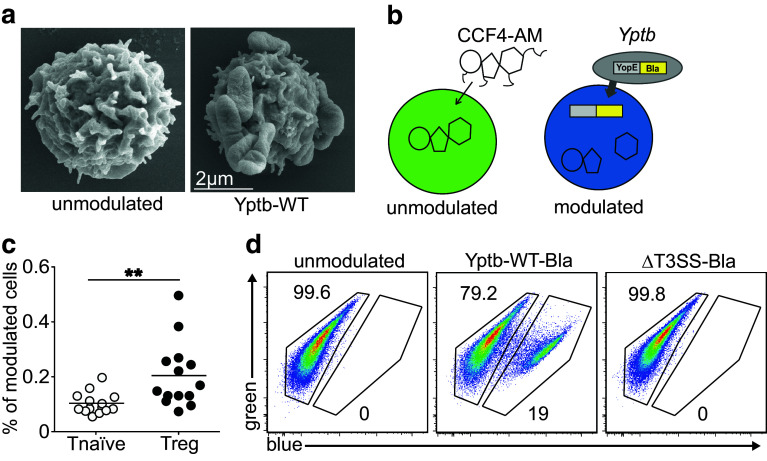
*Y. pseudotuberculosis* directly targets CD4^+^ T cell subsets. **a** Scanning electron microscopy of unmodulated and Yptb-WT-modulated naïve CD4^+^ T cells; representative pictures taken from one experiment. **b** Scheme of the β-lactamase reporter assay: upon Yop translocation into cells loaded with CCF4-AM, the β-lactamase-sensitive dye is cleaved, resulting in a fluorescence shift from* green* to* blue*. **c** *Scatter plot* depicts frequency of *Y. pseudotuberculosis*-modulated CD25^−^CD4^+^ naïve T cells and CD25^hi^CD4^+^ Tregs in mLNs assessed 3 days after intragastric infection of BALB/c mice with 2 × 10^9^ Yptb-WT-Bla. Data are pooled from two independent experiments, each with 6–7 mice per group. **p < 0.01. **d** Ex vivo-isolated naïve CD4^+^ T cells were co-cultured with Yptb-WT-Bla or ΔT3SS-Bla for 1 h or were left unmodulated as control. The frequencies of *Y. pseudotuberculosis*-modulated cells were analyzed by flow cytometry utilizing the β-lactamase reporter assay. Representative dot plots demonstrate the shift in fluorescence from* green* to* blue* in Yptb-WT-Bla-modulated cells (*middle panel*), or lack of blue cells in unmodulated and ΔT3SS-Bla-modulated cells (*left* and *right panels*, respectively)

Our hitherto existing results suggested that acute *Y. pseudotuberculosis* infection causes an alteration of the Th17-Treg balance by strongly affecting the cDC compartment. However, in addition to these indirect effects on T cell differentiation, more direct effects on T cell fate decisions are conceivable. Thus, we next asked whether *Y. pseudotuberculosis* could also directly interact with CD4^+^ T cells. At first, we performed field emission scanning electron microscopy analyses after co-culturing naïve CD4^+^ T cells with Yptb-WT in vitro and observed a direct attachment of *Yersiniae* to naïve T cells (Fig. 3a). In order to identify CD4^+^ T cell subsets being targeted by *Y. pseudotuberculosis*, we next applied a β-lactamase reporter assay [28, 29]. To this end, a Yptb-WT strain variant was generated via chromosomal integration of a YopE-β-lactamase fusion plasmid. This strain, named Yptb-WT-Bla, allows the assessment of Yop translocation into cells loaded with the cell permeable, β-lactamase-sensitive dye CCF4-AM [28, 29], resulting in a fluorescence shift from green to blue (Fig. 3b). In vitro co-culture of CD25^hi^CD4^+^ Tregs with Yptb-WT-Bla resulted in slightly higher translocation frequency as compared to naïve CD4^+^ T cells (data not shown). To monitor in vivo Yop translocation and to identify CD4^+^ T cell subsets being modulated by *Y. pseudotuberculosis*, BALB/c mice were infected sublethally with Yptb-WT-Bla. At day 3 p.i., the frequency of modulated cells among naïve CD4^+^ T cells and CD25^hi^CD4^+^ Tregs from mLNs was analyzed by flow cytometry. The β-lactamase reporter assay revealed that the frequency of in vivo-modulated Tregs, albeit being at a low level, was significantly higher when compared to naïve CD4^+^ T cells (Fig. 3c). To assess the role of the T3SS in modulating CD4^+^ T cells, a variant of the ΔyscS T3SS mutant strain [6], named ΔT3SS-Bla, was generated via chromosomal integration of the YopE-β-lactamase fusion plasmid. After in vitro co-culture of naïve CD4^+^ T cells with ΔT3SS-Bla, all modulated cells fully retained their green fluorescence, in contrast to Yptb-WT-Bla-modulated cells showing a substantial fraction of blue cells (Fig. 3d). In conclusion, the β-lactamase reporter assay enabled to demonstrate direct targeting of CD4^+^ T cell subsets, particularly Tregs, by *Y. pseudotuberculosis* in vivo. Moreover, the ΔT3SS-Bla strain proved to be helpful to further dissect alterations in T cell functional properties affected by the T3SS.

### *Yersinia* interferes with TCR-induced Ca^2+^ signaling and ERK phosphorylation within both naïve T cells and Tregs

**Fig. 4:**
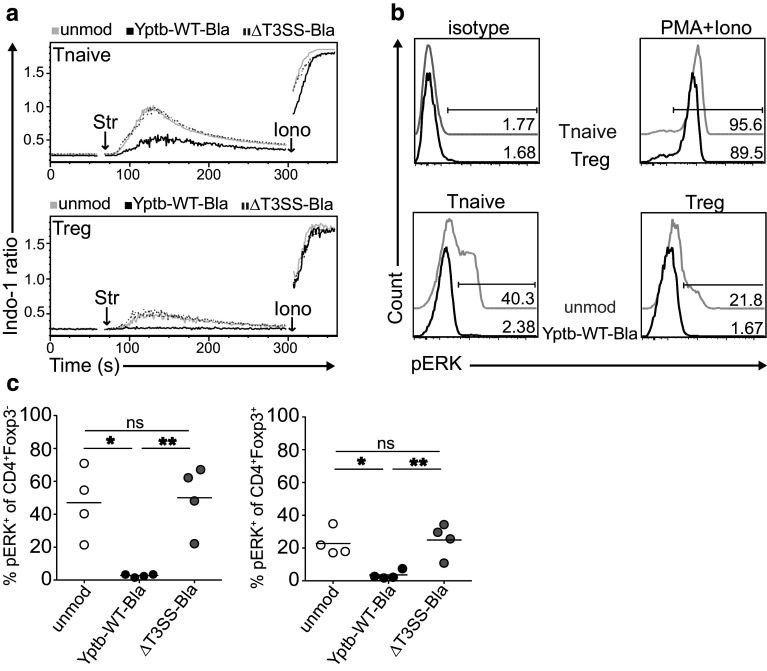
*Y. pseudotuberculosis* modulates naïve T cells and Tregs by interfering with TCR downstream signaling pathways. CD4^+^ T cells were enriched from secondary lymphoid organs of Foxp3^hCD2^ mice and co-cultured with Yptb-WT-Bla or ΔT3SS-Bla for 1 h. Subsequently, TCR crosslinking of anti-CD3/CD28 pre-decorated cells was induced by addition of streptavidin (Str). **a** Intracellular Ca^2+^ flux was assessed in Foxp3^hCD2−^ naïve T cells and Foxp3^hCD2+^ Tregs by flow cytometry using the cell permeable Indo-1 AM dye. Ca^2+^ flux in response to the positive control ionomycin (Iono) was equivalent between all groups. *Histograms* show representative results of three independent experiments. **b** Phosphorylation of ERK was assessed 1.5 min after TCR crosslinking by intracellular pERK staining and flow cytometry in Foxp3^hCD2−^ naïve T cells and Foxp3^hCD2+^ Tregs. Representative histograms depict pERK expression of gated cells from indicated groups, and numbers indicate frequencies of pERK^+^ cells. The region was set according to isotype control staining of unmodulated cells (*upper left*). Stimulation of cells with PMA plus Iono served as positive control (*upper right*). **c** *Scatter plots* summarize frequencies of pERK^+^ cells among Foxp3^hCD2−^ naïve CD4^+^ T cells (*left*) and Foxp3^hCD2+^ Tregs (*right*). Data are pooled from four independent experiments. *Ns* not significant; *p < 0.05; **p < 0.01

As translocation of Yops can modulate host cell signaling responses [4], we next aimed to unravel downstream events of TCR signaling influenced by *Y. pseudotuberculosis*. First, we analyzed intracellular Ca^2+^ flux in CD4^+^ T cells stimulated via their TCR by using Indo-1 AM cell permeable dye as a rapid and sensitive measure of TCR activation. TCR crosslinking was induced via the addition of streptavidin to cells pre-decorated with biotinylated anti-CD3/CD28 antibodies. Similar to what has been reported before [30], TCR-triggered Ca^2+^ signaling of naïve CD4^+^ T cells was profoundly higher as compared to Foxp3^+^ Tregs (Fig. 4a). Interestingly, after co–culture with Yptb-WT for 1 h, naïve CD4^+^ T cells showed a strong reduction in Ca^2+^ signaling, while Ca^2+^ flux into Foxp3^+^ Tregs even was completely blocked. This interference with TCR-induced Ca^2+^ signaling was strictly dependent on the T3SS as modulation of CD4^+^ T cells with ΔT3SS-Bla did not alter Ca^2+^ signaling within both CD4^+^ T cells and Foxp3^+^ Tregs (Fig. 4a), suggesting that translocation of Yops critically contribute to the modulation of TCR downstream signaling. Secondly, we measured TCR-induced phosphorylation of ERK (pERK), a more specific indicator of TCR activation, after the modulation of naïve CD4^+^ T cells with *Y. pseudotuberculosis*. Kinetic studies (0–5 min after TCR crosslinking) revealed that the peak of ERK phosphorylation occurred after 1.5 min (data not shown), and this time point was chosen for all further analyses. In accordance with published data [30], unmodulated naïve CD4^+^ T cells showed a higher frequency of pERK^+^ cells as compared to unmodulated Foxp3^+^ Tregs, while both cell types showed strong pERK activation upon stimulation with PMA and ionomycin (Fig. 4b). Upon modulation with Yptb-WT, ERK phosphorylation was completely abrogated in both naïve CD4^+^ T cells and Foxp3^+^ Tregs, and again this interference with TCR-induced signaling was strictly dependent on the T3SS as modulation of CD4^+^ T cells with ΔT3SS-Bla did not result in a reduced ERK phosphorylation in any of the two T cell subsets (Fig. 4b, c). Together, our data suggest that *Y. pseudotuberculosis* can directly interfere with TCR downstream signaling in both naïve CD4^+^ T cells and Foxp3^+^ Tregs, most likely through translocation of Yops in a T3SS-dependent manner.

### Modulation of naïve T cells with *Y. pseudotuberculosis* supports the differentiation of Th17 cells, but disturbs de novo induction of Foxp3^+^ Tregs

**Fig. 5:**
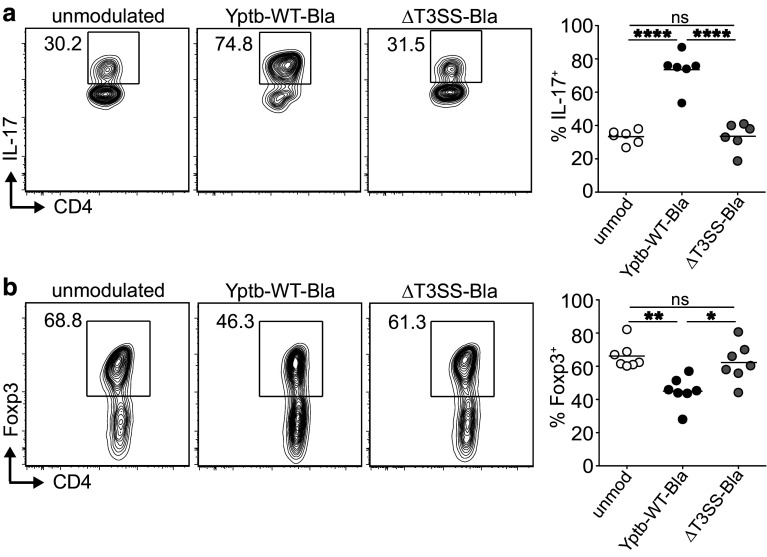
In vitro modulation of naïve T cells with *Y. pseudotuberculosis* results in increased differentiation of Th17 cells and decreased de novo induction of Foxp3^+^ Tregs. Naïve CD4^+^ T cells were enriched from secondary lymphoid organs of Foxp3^hCD2^ mice and co-cultured with Yptb-WT-Bla or ΔT3SS-Bla for 1 h, or were left unmodulated as control. Subsequently, modulated T cells were cultured under Th17-polarizing or Treg-inducing conditions for 6 or 4 days, respectively, and IL-17 or Foxp3 expression was assessed by flow cytometry at the end of the cultures. Representative *dot plots* show expression of IL-17 (**a**) and Foxp3 (**b**) in cells from indicated cultures. *Numbers* indicate frequencies of cells in gates. *Scatter plots* summarize frequencies of IL-17^+^ (**a**) and Foxp3^+^ (**b**) cells from indicated cultures. Data are pooled from six (**a**) or seven **b** independent experiments, and means of technical replicates are depicted. *p < 0.05; **p < 0.01; ****p < 0.0001

Having demonstrated that *Y. pseudotuberculosis* can directly modulate early events of TCR downstream signaling, we next asked which consequences this modulation might have on de novo generation of Th17 cells and Foxp3^+^ Tregs. To this end, ex vivo-isolated naïve CD4^+^ T cells were co-cultured with Yptb-WT-Bla or ΔT3SS-Bla for 1 h, followed by gentamicin treatment to kill living bacteria. Subsequently, modulated naïve CD4^+^ T cells were stimulated in vitro in an antigen-presenting cell (APC)-free system under polarizing conditions. Stimulation of Yptb-WT-Bla-modulated naïve CD4^+^ T cells under Th17-polarizing conditions resulted in a significantly enhanced frequency of IL-17^+^ cells at day 6 of the culture when compared to differentiation of unmodulated naïve CD4^+^ T cells (Fig. 5a), although expression of the lineage specification transcription factor RORγt remained unaffected (Supplementary Fig. S3). Importantly, this enhanced Th17 differentiation was strictly dependent on the T3SS as modulation of CD4^+^ T cells with ΔT3SS-Bla did not result in an increased frequency of Th17 cells.

The opposite was observed when Yptb-WT-Bla-modulated naïve CD4^+^ T cells were stimulated under Treg-inducing conditions. Here, we saw a significantly reduced frequency of Foxp3^+^ Tregs at day 4 of the culture when compared to differentiation of unmodulated naïve CD4^+^ T cells (Fig. 5b). Also this disturbance of in vitro Treg differentiation was dependent on the T3SS as no impact could be observed upon modulation of naïve CD4^+^ T cells with ΔT3SS-Bla (Fig. 5b). Importantly, *Y. pseudotuberculosis* did not modulate the capacity of naïve CD4^+^ T cells to differentiate into IFN-γ-producing cells as no difference in frequencies of IFN-γ^+^ cells was observed in Th0 or Th1 cultures when Yptb-WT-Bla-modulated naïve CD4^+^ T cells were directly compared with unmodulated cells (Supplementary Fig. S4).

Together, these in vitro data suggest that the direct T3SS-dependent modulation of TCR downstream signaling within naïve CD4^+^ T cells alters their differentiation potential, resulting in a skewing from suppressive Foxp3^+^ Tregs to proinflammatory Th17 cells.

## Discussion

In the intestine, the balance between effector and regulatory pathways needs to be tightly controlled in order to maintain immune homeostasis and efficiently combat infections. Therefore, the ability of CD4^+^ T cells to change their phenotype and to acquire special functional properties might be critical during acute infection with enteropathogenic *Y. pseudotuberculosis*. Here, we report that *Yersiniae* selectively disrupt the balance between Tregs and Th17 cells, towards an increased differentiation of proinflammatory Th17 cells and a reduction in the generation of immunosuppressive Tregs, whereas the frequency of IFN-γ-producing Th1 cells or IL-10-producing T cells remained largely unaltered. The *Yersinia*-mediated support of proinflammatory responses is surprising as pathogenic bacteria usually promote Treg expansion, enabling their long-term survival in the host and establishment of chronic infection [31, 32]. However, *Yersiniae* rather impair efficient de novo Treg induction within gut-draining mLNs, potentially favoring systemic dissemination of the pathogen [17]. Furthermore, increased de novo generation of Th17 cells could support the pathogen in establishing an inflammatory environment via altering microbiota composition, thereby favoring colonization of *Y. pseudotuberculosis*, similar to changes instigated by *Citrobacter rodentium* and *Salmonella* infections [33, 34]. On the other hand, the relatively late appearance of endogenous Th17 cells argues against a microbiota-induced ‘innate-like’ character, which had been reported previously [35], but rather suggests that these Th17 cells are pathogen-specific. Thus, our data represent first evidence that *Yersiniae* disrupt the Th17-Treg balance, which might be a critical strategy of the pathogen in establishing acute infection.

To better understand how *Yersiniae* fine-tune the CD4^+^ T cell compartment, we characterized DC subsets within mLNs during acute infection. It had been shown before that inflammatory conditions within the gut not only alter the distribution of DCs [36, 37], but also negatively affect the tolerogenic CD103^+^ subset [25], which is known to efficiently promote de novo generation of Foxp3^+^ Tregs [23, 26]. Therefore, we first hypothesized that *Y. pseudotuberculosis* indirectly affects T cell differentiation via modulation of DC subsets within mLNs. Indeed, the strongly decreased frequency of tolerogenic CD103^+^CD8^+^ DCs could account for reduced Treg induction [23, 24, 38], although it cannot be formally excluded that the reduced frequency of CD103^−^CD8^+^ DCs, which most probably belong to resident DCs [27] and for which a tolerogenic phenotype has been described before [39], are also responsible. In contrast, expansion of the CD103 DC subset might be responsible for increased differentiation of Th17 cells [24, 40]. Interestingly, in a recently published study chronic infection with *Y. pseudotuberculosis* only resulted in a decreased frequency of CD103^+^CD11b^+^ DCs, without affecting any other DC subsets [9]. This is in contrast to data from the present study, where during the acute phase of infection, frequencies of both CD103^+^ and CD103^−^ DCs were altered by enteropathogenic *Yersiniae*, and most likely these changes jointly contribute to the establishment of a strong inflammatory response. Moreover, increased CD86 expression levels of the latter DC subset demonstrate their highly activated status and suggest the possible involvement of Toll-like receptors (TLRs) in creating a shift towards Th17-dominated immune responses [41–43]. However, it still needs to be investigated whether TLR agonists of *Y. pseudotuberculosis* contribute to the increased Th17 differentiation.

Nonetheless, *Yersiniae* cannot only affect the DC compartment. Using the β-lactamase reporter system, we could demonstrate that upon infection via the natural route *Yersiniae* can directly modulate CD4^+^ T cells within infected mLNs, extending previous findings from a systemic infection model [29]. Interestingly, within the CD4^+^ T cell population, Tregs were preferentially modulated when compared to naïve T cells. The increased translocation rate of Yops into Tregs can be explained by the binding of *Y. pseudotuberculosis* invasin to β1 integrin VLA4 [44], which is expressed at higher levels in Tregs as compared to naïve T cells [45].

When dissecting the mechanism of *Yersiniae*-dependent Treg modulation and alteration of naïve T cell differentiation, we observed a stark decline of Ca^2+^ mobilization after TCR triggering for naïve CD4^+^ T cells, while Ca^2+^ signaling within Foxp3^+^ Tregs was even fully abrogated. In line with reduced Ca^2+^ mobilization, phosphorylation of ERK was strongly abolished in both naïve CD4^+^ T cells and Foxp3^+^ Tregs. Since ΔT3SS-Bla affected neither Ca^2+^ signaling nor ERK phosphorylation within both cell types, our findings indicate that direct modulation of T cells requires the presence of the T3SS. The importance of Yop translocation was also suggested from results of the APC-independent, in vitro T cell differentiation assay, where increased frequencies of Th17 cells and reduced de novo Treg induction were observed only upon co-culture of naïve T cells with Yptb-WT and not with T3SS-deficient *Yersiniae*. Among Yops, the tyrosine phosphatase YopH is known to interfere with early T cell receptor signaling events, either via direct phosphorylation of signaling molecules [46–48], or by binding to adaptor proteins, which then target them to signaling complexes [48, 49]. Thus, YopH might be responsible for the reduced Ca^2+^ mobilization and ERK phosphorylation we observed in CD4^+^ T cell subsets being modulated by *Y. pseudotuberculosis*. Moreover, ERK activity in Jurkat T cells had been restored by addition of a YopH inhibitor [50], further arguing for the involvement of YopH in the reduction of ERK phosphorylation of both naïve CD4^+^ T cells and Foxp3^+^ Tregs. Additionally, the interference of YopH with TCR signaling could also lead to reduced secretion of IL2 [47, 51]. Since IL-2 is a factor critically required for induction of Tregs and at the same time can prevent Th17 differentiation [52, 53], it is tempting to speculate that *Y. pseudotuberculosis*mediated alterations in IL-2 production also contribute to the skewing from Tregs to Th17 cells, and the lower frequency of de novo-induced Tregs might enable enhanced generation of Th17 cells. Yet, whether YopH is the master regulator of *Y. pseudotuberculosis*-mediated modulation of T cell differentiation or other mechanisms contribute to the fine-tuning of the closely related transcriptional regulation of Tregs and Th17 cells [54, 55] remains to be elucidated. In this context, it will be also interesting to dissect why modulation of naïve CD4^+^ T cells with Yptb-WT-Bla did only affect expression of the cytokine IL-17 in Th17-polarizing cultures, but not the expression of the Th17 lineage specification factor RORγt, although under Treg-inducing conditions a severe impact on the expression of the Treg lineage specification factor Foxp3 could be observed.

Taken together, the present study implicates a critical role of CD4^+^ T cell subsets in the pathomechanism of acute *Y. pseudotuberculosis* infection, showing that this enteropathogen favors the generation of Th17 cells, and in parallel leads to a decline in Treg induction. Our data provide evidence that *Y. pseudotuberculosis* interferes with TCR signaling in both Foxp3^+^ Tregs and naïve CD4^+^ T cells, thereby directly modulating T cell-mediated immune responses. Efforts to understand the precise pathomechanism of gastric infections could permit the development of future therapeutics for an efficient modulation of the immune system.

### Electronic supplementary material

Below is the link to the electronic supplementary material.

Supplementary material 1: Endogenous T helper cell subsets in mLNs of Yptb-WT-infected mice. BALB/c mice were infected with 2 × 10^8^ Yptb-WT, and the frequency of cytokine-producing endogenous CD4^+^ T cells was analyzed at indicated time points p.i. by flow cytometry. Graphs depict frequencies of (**a**) IFN-γ-, (**b**) IL-10- and (**c**) IL-17-producing CD4^+^CD3^+^ T cells in mLNs of mice infected with Yptb-WT. PBS-treated mice were taken as uninfected controls. Data are pooled from two independent experiments with 2–8 mice per group. *p < 0.05. (EPS 614 KB)

Supplementary Figure S2: Characterization of cDC compartment in mLNs of Yptb**-**WT-infected mice. BALB/c mice were infected with 2 × 10^8^ Yptb-WT, and at day 5 p.i. the frequency and absolute number of cDCs within mLNs was determined by flow cytometry. (**a**) Representative dot plots depict MHCII^hi^CD11c^hi^ cDCs among Lin^-^(CD3^−^CD19^−^CD49b^−^B220^−^) cells in mLNs of uninfected control mice or in mLNs of mice infected with Yptb-WT. Numbers indicate frequencies of cells in gates. (**b**) Scatterplot summarizes frequencies of MHCII^hi^CD11c^hi^ cDCs among Lin^−^ cells within mLNs of indicated groups. Data are pooled from two independent experiments with 3–7 mice per group. ***p < 0.001. (**c**) Scatterplot depicts absolute number of MHCII^hi^CD11c^hi^ cDCs within mLNs of indicated groups. Data are pooled from two independent experiments with 3–7 mice per group. Ns, not significant. (EPS 747 KB)

Supplementary Figure S3: In vitro modulation of naïve CD4^+^ T cells with *Y. pseudotuberculosis* and culture under Th17 polarizing conditions does not affect RORγt expression. Naïve CD4^+^ T cells were enriched from secondary lymphoid organs of Foxp3^hCD2^ mice and co-cultured with Yptb-WT-Bla and ΔT3SS-Bla for one hour, or were left unmodulated. Subsequently, modulated T cells were cultured under Th17-polarizing conditions for six days, and RORγt expression was assessed by flow cytometry at the end of the cultures. Representative dot plots from two independent experiments show expression of RORγt in cells from indicated cultures. Numbers indicate frequencies of cells in gates. (EPS 624 KB)

Supplementary Figure S4: In vitro modulation of naïve CD4^+^ T cells with *Y. pseudotuberculosis* does not affect IFN-γ production of cells cultured under Th0 or Th1 conditions. Naïve CD4^+^ T cells were enriched from secondary lymphoid organs of Foxp3^hCD2^ mice and co-cultured with Yptb-WT-Bla for one hour, or were left unmodulated as control. Subsequently, modulated T cells were cultured under Th0-polarizing or Th1-inducing conditions for five days, and IFN-γ expression was assessed by flow cytometry. Scatterplot summarizes frequencies of IFN-γ^+^ cells from indicated cultures. Data are pooled from two independent experiments, and means of technical replicates are depicted. Ns, not significant. (EPS 518 KB)

## Abbreviations

*Y. pseudotuberculosis*: *Yersinia pseudotuberculosis*
mLN: Mesenteric lymph node
T3SS: Type III secretion system
Yops: Yersinia outer proteins
DC: Dendritic cell
Treg: Regulatory T cell
TCR: T cell receptor
Yptb-WT: Wild-type *Y. pseudotuberculosis*
Ova: Ovalbumin
APC: Antigen-presenting cell
TLR: Toll-like receptor
